# “Broadband” Bioinformatics Skills Transfer with the Knowledge Transfer Programme (KTP): Educational Model for Upliftment and Sustainable Development

**DOI:** 10.1371/journal.pcbi.1004512

**Published:** 2015-11-19

**Authors:** Emile R. Chimusa, Mamana Mbiyavanga, Velaphi Masilela, Judit Kumuthini

**Affiliations:** 1 Computational Biology Group, Department of Integrative Biomedical Sciences, Institute of Infectious Disease and Molecular Medicine, University of Cape Town, Cape Town, South Africa; 2 Centre for Proteomic and Genomic Research, Cape Town, South Africa; Ontario Institute for Cancer Research, CANADA

## Abstract

A shortage of practical skills and relevant expertise is possibly the primary obstacle to social upliftment and sustainable development in Africa. The “omics” fields, especially genomics, are increasingly dependent on the effective interpretation of large and complex sets of data. Despite abundant natural resources and population sizes comparable with many first-world countries from which talent could be drawn, countries in Africa still lag far behind the rest of the world in terms of specialized skills development. Moreover, there are serious concerns about disparities between countries within the continent. The multidisciplinary nature of the bioinformatics field, coupled with rare and depleting expertise, is a critical problem for the advancement of bioinformatics in Africa. We propose a formalized matchmaking system, which is aimed at reversing this trend, by introducing the Knowledge Transfer Programme (KTP). Instead of individual researchers travelling to other labs to learn, researchers with desirable skills are invited to join African research groups for six weeks to six months. Visiting researchers or trainers will pass on their expertise to multiple people simultaneously in their local environments, thus increasing the efficiency of knowledge transference. In return, visiting researchers have the opportunity to develop professional contacts, gain industry work experience, work with novel datasets, and strengthen and support their ongoing research. The KTP develops a network with a centralized hub through which groups and individuals are put into contact with one another and exchanges are facilitated by connecting both parties with potential funding sources.

This is part of the *PLOS Computational Biology* Education collection.

## Introduction and Background

An overwhelmingly large number of African countries fall below average on the standard indices of science and technology capacity [1, 2]. A shortage of technical skills and relevant expertise is often the primary obstacle to sustainable development in Africa. The legislative and infrastructural environment in many African countries is not conducive to research [2, 3]. In addition, over the years, Africa has witnessed a steady loss of university staffs, which has led to low scientific research output and inadequate preparation of the next generation of African biomedical and life scientists [3, 4]. Further impediments include limited internet connectivity, lack of computational resources, and substandard research facilities. This is particularly problematic in countries whose aim is to implement the transition from resource-based to knowledge-fuelled economic growth. Consequently, individuals who receive international training in highly resourced environments usually struggle to function or implement their learnt expertise and knowledge upon return to their often resource-poor home environments. Short-term training programmes are useful but have proved to be unsustainable and costly, since knowledge and skills are not retained over time [2]. The biomedical industry is a pillar of socioeconomic development in developed nations and is seen as the catalyst of economic growth in Africa against the backdrop of examples set by China, India, and Brazil [5, 6]. Various initiatives, including the Global Organization for Bioinformatics Learning, Education and Training (GOBLET, http://mygoblet.org/), the International Society for Computational Biologists (ISCB), H3ABioNet (http://www.h3abionet.org/), and H3Africa (http://h3africa.org/), amongst others, are underway to strengthen bioinformatics research capacity and education in Africa [2, 6, 7]. Such organizations, if well-designed and focused, are likely to foster biomedical and agricultural biotechnology research and project implementation [2, 7–9]. However, current development capacity in biomedical research is largely based on input from a few isolated senior scientists in Africa and abroad.

### Key African Challenges in Critical Mass Development in Bioinformatics

There are various challenges that are encountered within the critical mass development of bioinformatics on the African continent. A shortage of relevant bioinformatics skills has led to ongoing discussions about implementing the appropriate curriculum within the bioinformatics field in Africa [8, 10]. The study of bioinformatics is complex and consists of multiple disciplines essential to biomedical and life science research [8]. Due to the diverse backgrounds of students, it is challenging to improve capacity within bioinformatics and computational biology fields; when students have had a limited exposure to the subject field, there is often a limited grasp [8–10]. This lack of capacity impacts socioeconomic growth, disabling African countries from taking advantage of a knowledge-based economy, compared to other developing countries like China and Brazil [6]. Another confounding factor is limited job opportunities in the field of bioinformatics. This results in graduates opting to apply their technical skills in other sectors of the economy where analytical skills are highly sought after.

### Increase in Bioinformatics Demand

Bioinformatics is arguably a recent field of study, but it has developed to a substantial level of importance in the past two decades. The amount of biological data generated by sequencing projects of various species, genome variation studies, and other large data “omics” studies present challenges; demand outstrips supply, and capacity to interpret this complex data is limited in Africa. Ozlem [8, 10–13] argues that in recent decades there has been substantial advancement in the modernization of techniques and established approaches within life sciences, and that continuous development of capacity within the field of computational biology is required.

### African Genomic Variation

Researchers in these fields have been interested in studying people in Africa, known as the origin of modern humans, in order to understand the original source of all human genetic diversity [14, 15]. Africa faces a heavy, growing burden of non-communicable diseases [14,16]. The unique environmental impact on medical genetics, as well as genetic factors that contribute to diseases in most African populations, are challenges for African researchers [14,15]. Over the last few years there has been a remarkable growth of African genomic data, starting with the systematic re-sequencing of specific genomic regions [15], followed by next-generation sequencing of the entire genome of an African individual [17,18], leading on to the 1000 Genomes Project (http://www.1000genomes.org/) and the African Genome Variation Project [19]. From a statistical genetics perspective, the high levels of genetic diversity and the burden of complex diseases in African populations offer both challenges and opportunities for genomic analysis [14–16].

While the bioinformatics genomic study of African ethnic groups has commenced, it is still in its infancy and is mostly conducted overseas due to the shortage of practical skills within the research community in Africa. However, the Knowledge Transfer Programme (KTP) aims to match African computational biomedical research questions with knowledgeable experts. This has great benefits, not only for sharing knowledge but also for sustaining further collaborations between local African researchers and the experts.

### Lack of Infrastructure As an Emerging Economic Income Continent

African countries share common challenges with other developing countries; however, infrastructure remains central to the problem [6, 20]. The lack of reliable internet connectivity inhibits the growth of bioinformatics in Africa. This challenge creates difficulties in administering certain bioinformatics processes that depend on reliable internet connectivity [6–8]. As a result, most bioinformatics training takes place in shared facilities, either in a university or other tertiary institution [6–8]. Consequently, facilities tend to be oversubscribed and in short supply, while conflicts also arise with other academic fields, such as information technology, which need the facilities for coursework. Overcrowding of multiple software applications on the system provides very little space for related bioinformatics software to support any undertaken studies [21, 22]. Although faced with these challenges, support programmes can mitigate the problem. An example of such an initiative is H3ABioNet, which contributes significantly to project funding and provides training equipment (e.g., eBiokits and Visyo) to local organizations to facilitate quality training and mitigate some of these challenges [20, 22].

### The Induced Brain Drain of Bioinformatics Skills in Africa

A substantial number of African scientists, including bioinformaticians, go abroad because of competitive salaries, better job opportunities, and well-resourced institutions. This creates a strain on local, skilled bioinformaticians while the need for bioinformatics capabilities continues to increase [20, 21, 23]. As a result, the brain drain of bioinformatics skills in Africa is alarming; numbers continue to decline in the science and technology statistics averages [20–22]. Moreover, there is a steady decline in leading African experts, who have resorted to collaborating with organizations abroad (mostly in the United States and Europe) to complete research and make use of advanced facilities [1, 8].

Conventional methods of training are initiated by sending researchers to overseas organizations; upon return, they are required to share their learnt knowledge in Africa. However, a lack of mentoring has left many students unable to partake in the field [6, 7]. Although various initiatives are underway to strengthen bioinformatics education in Africa [2, 5, 6, 20], current development capacity in biomedical research is largely based on input from a few isolated senior scientists in Africa and abroad. In addition, disparity across the continent regarding capacity-building in bioinformatics is still a serious concern.

The general approach to international development suggests that developing countries should self-identify their problems and collaborate with developed countries to mitigate these problems [8, 20, 23]. Bioinformatics exists in a rapidly evolving environment. This growth necessitates the equal growth of bioinformaticians to efficiently contribute to ongoing study [2, 20, 23, 24]. If initiatives could be well defined and implemented, they would foster biomedical and life sciences research. However, development in biomedical research is largely based on input from a few isolated senior scientists from Africa, many of whom are based abroad [8, 20, 22–24].

This article describes the KTP, an initiative supported by the Centre for Proteomic and Genomic Research (CPGR) and the NIH-funded H3ABioNet project to strengthen bioinformatics capability-building at local institutions in Africa where life scientists are in need of skilled bioinformaticians. The KTP is an online matchmaking platform that brings together seekers of bioinformatics expertise with experts who are willing to transfer their knowledge in a project-based fashion. These experts physically assist with the development and monitoring of local trainers and trainees through a “train-the-trainer” component associated with every project proposed by the knowledge-seeker.

**Fig 1:**
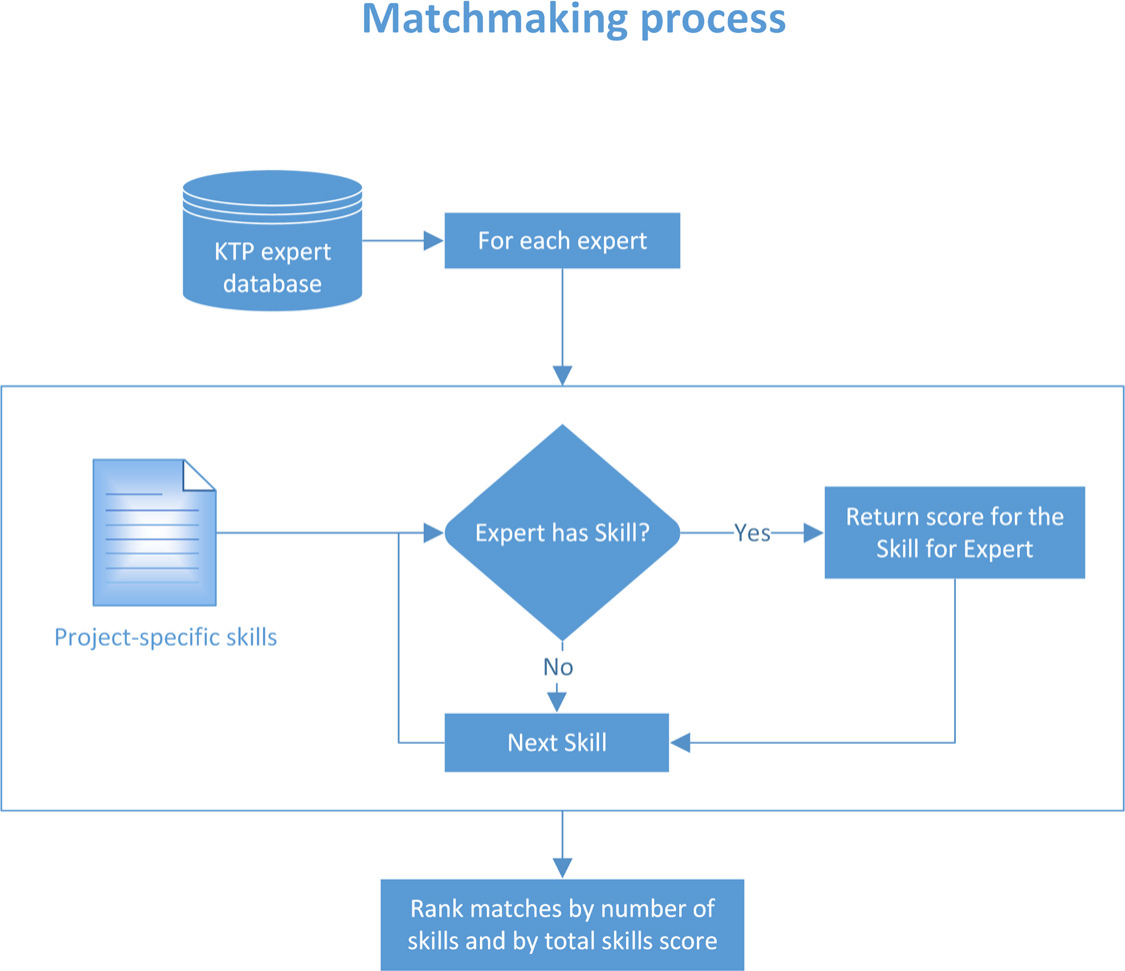
Process map of the matchmaking platform.

The KTP and its Review Committee (RC) and Scientific Advisory Committee (SAC) (see Table 1) facilitate the process by identifying and evaluating experts (knowledge providers), projects (proposed by knowledge-seekers, also known as principal investigators [PIs]), and training requirements for potential research associates and trainees, respectively (Fig 1 and S1 Text). Instead of travelling overseas, experts are brought in locally to conduct high-quality research agendas and work on relevant projects, through which the transfer of knowledge and skills is achieved naturally (Fig 1). One of the advantages to this approach is minimized travel and accommodation expenditure. The programme’s success is monitored by the number of local junior and senior bioinformatics trainers created, the number of trainees trained, the number of experts in the expert database, and the number of projects supported (S1 Text). Importantly, the KTP is highly linked to international organizations, including the European Molecular Biology Network (EMBnet), GOBLET, Golden Helix Foundation, and H3ABioNet, amongst others, which contribute significantly toward the goal of increasing bioinformatics capacity and knowledge expansion in Africa, which is essential to the KTP’s objectives (S1 Text).

**Table 1:** Description of the KTP’s stakeholders and benefits offered by the KTP to each of the stakeholders.

Stakeholders	Description	Benefits
Knowledge-seeker/Principal Investigator (PI)	Any scientist at a university or in a private or public organisation with a need for bioinformatics skills development in a particular project.	Access to expert support for running the project; equipping of staff to effectively work on the project; access to KTP facilities and resources for executing the project; reduction in project running costs as a result of collective KTP funding; quick and easy identification of support from experts in the KTP database; future international collaboration; capacity developed locally.
Trainee/Associate	Any scientist, researcher, student, or employee in need of bioinformatics training. A subset of trainees will form a local contingent of potential future African trainers.	Faster turnaround time for the transfer of knowledge; access to all the trainings offered locally; opportunity to establish collaboration links with fellow associates or experts on the project; direct implementation of the acquired knowledge.
Expert	Researcher, professional, or field application specialist with proven expertise in a relevant area of computational biology, bioinformatics, or biostatistics.	International experience and exposure to an African setting for doing research; opportunity to share and transfer knowledge as a project expert and/or trainer; novel dataset or problem; potential publication or patent; potential collaboration.
Hosting Institution	Institution associated with the PI that will provide the facilities for the execution of a KTP project and/or a KTP training session. In addition, there are administrative structures to manage the KTP as a whole.	Increased profile for the hosting institution; contribution to research and development in Africa.
Scientific Advisory Committee (SAC)	Selected group of researchers and professionals with relevant expertise, assembled to safeguard the quality of project proposals and the matchmaking process between the experts and the project.	International experience and exposure to an African setting for doing research; social upliftment in the field of expertise; provides a level of personal satisfaction.
Review Committee (RC)	Selected group of researchers who review and assess the bioinformatics expert applications to ensure the quality of the expert base.	International experience and exposure to African setting for doing research; social upliftment in the field of expertise; provides a level of personal satisfaction.

### Matchmaking Platform

Reviewing activities of the experts includes assessing and validating their skills, experience, and contribution to the scientific community before admitting them into the system and accepting them as either junior or senior experts. S1 Fig illustrates the flow of information during the expert review process (more details in S1 Text). Similarly, the registration of the project includes relevant information about the project, a work package (proposing times and deliverables), and the set of required skills for this project (see S2 Fig and S1 Text for more details). A project PI identifies certain expertise from the KTP skills matrix tree format, adopted from the skills ontology of *PLOS Computational Biology*. The KTP matchmaking platform (Fig 1) identifies the most suitable expert from the KTP expert database, based on the project requirements submitted by the PI. Based on the number of matching skills requested for a project and the level of skill(s) an expert has, candidates are short-listed and matchmaking is undertaken (see S1 Text for more details). Secondly, the SAC ensures the project is feasible within the timeframe and can be achieved by the selected expert(s). All the expert applications, the review process, and the project selection process are streamlined within the KTP system through an online application process.

An arbitrary scoring system is used to quantify and qualify the skills provided by the individual applying to be an expert (details in S1 Text). In order to manage all the reasoning behind the KTP’s different processes and to retain audit trails (Figs 1, S1 and S2), a web system was designed and implemented (http://ktp.cpgr.org.za/). The KTP is organized around six main stakeholders, whose benefits are shown in Table 1.

## Discussion

Education and skills training are critical to achieving competitive advantage. The level of workforce skills needs to be constantly updated. Additionally, workforce requirements have changed; employees are required to have skills enabling them to use information to generate knowledge, to engage in collaborative problem-solving, to make decisions, to be self-driven and organized, and to work independently. These are all 21st century skills, which are rarely covered by formal school curricula [24–28]. Therefore, preparation is required to create a critical mass with essential skills for the development of expert decision-making and metacognitive strategies, i.e., the ability to proceed when no standard approach seems applicable [8, 26–28]. However, ongoing efforts are in place with a strong focus on skills enhancement and sustainable development in Africa [26]. Africa has a window of opportunity to broaden and expand the economic benefits derived from their natural capital. However, the lack of specialized expertise has proven to be a major bottleneck, obstructing the potential for more well-paid jobs and home-grown knowledge [1, 6, 23, 27]. This is a common obstacle also faced within the biotechnology field, in which the bioinformatics skills gap needs to be addressed in order to attend to the trend of health expenditures that are fast outgrowing the gross domestic product (GDP). Much of the obstacle is also due to the unique population genetic structure present in many countries in Africa.

We have proposed a systematic yet customizable approach to building bioinformatics capacity in Africa in a more sustainable fashion. The KTP’s model is built on the premise that researchers with desirable skills are invited to join local research groups for six weeks to six months, instead of individual researchers travelling to overseas destinations for training. We postulate that visiting experts will pass on their skills and knowledge to multiple people simultaneously. This increases the efficiency of the concomitant knowledge transfer within the context of a specific project. In return, the visiting researcher is given the opportunity to develop professional contacts, gain work experience in a different geographical and cultural setting, and work on scientific problems and data unique to Africa. In addition, the local project will gain from the expert’s involvement by building a knowledge base and exposure to skills from a different environment. This translates into more efficient project management, completion within shorter timelines, the sustaining of future collaboration, and a subsequent reduction of costs.

The KTP is complementary to other channels for training in bioinformatics and is a tool and catalyst of “omics” research, with the goal of long-term capacity development and sustainability for local institutes and companies (more details in S1 Text). The KTP is a platform for solving the problem of the shortage in skills within bioinformatics and its application in the “omics” field. Therefore, the KTP serves as a catalyst for knowledge transfer and skills development, undertaken by experts and trainees in a project-based fashion in Africa. As a complement to existing conventional teaching and training methods, the KTP enables double cognitive apprenticeship, which refers to direct instruction for cognitive and technical skills, followed by project-based learning in a group in the real world of work. This is supported by trainers and more able peers in response to the increasing demand for the level of bioinformatics skills required for the development of critical mass [29].

## Conclusion

Significant skills shortages exist both in terms of numbers and quality, particularly within the science, technology, engineering, and mathematics (STEM) fields [11, 12, 28].

The KTP aims to develop a network with a centralized hub through which knowledge seekers from African institutes or companies and knowledge providers or experts from around the world are put into contact with one another, and exchanges of skills and knowledge are facilitated through an online KTP system. The focus of the programme is to develop and transfer hands-on experience in analysing data from various current proteomic, genomic, and other platforms, using cutting-edge techniques, analysis methods, tools, pipelines, and standards. The long-term benefits include the acquisition of knowledge and the creation of a pool of local experts and trainers who can, in turn, amplify this knowledge in Africa. The KTP provides an option for members to apply as an associate project member or expert and to propose projects or take part in training activities hosted under the KTP. The KTP has already successfully implemented projects and trainings, and our expert database has started to grow and receive good feedback.

## Supporting Information

S1 Fig KTP's expert application process. (TIF)

S2 Fig KTP's project application process. (TIF)

S1 Text Supplementary information. (DOCX)
