# Supplementary figures and images for: “Broadband” Bioinformatics Skills Transfer with the Knowledge Transfer Programme (KTP): Educational Model for Upliftment and Sustainable Development
Source: PLoS Comput Biol. 2015 Nov 19;11(11):e1004512. doi: 10.1371/journal.pcbi.1004512 (PMC4652891; doi:10.1371/journal.pcbi.1004512)

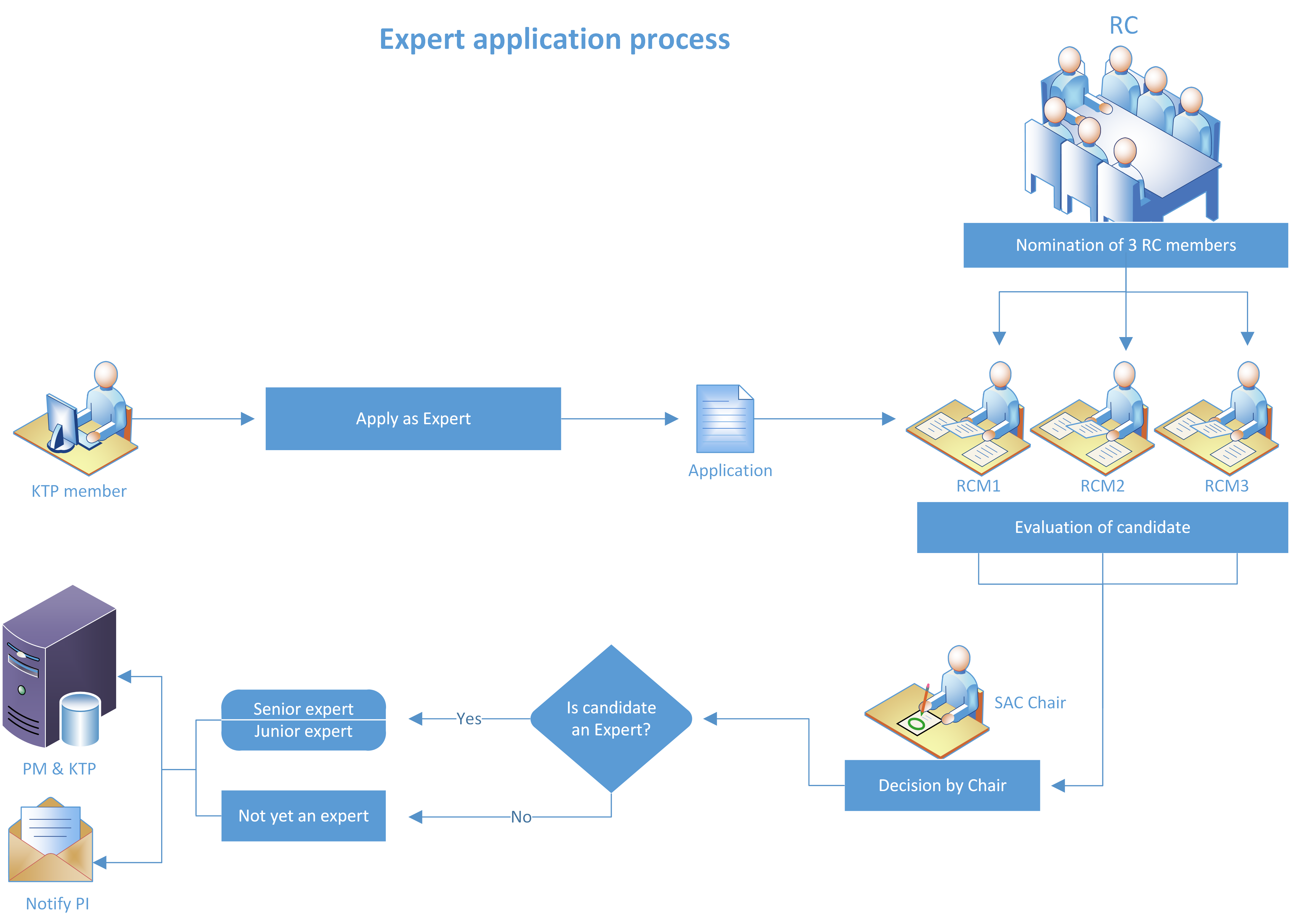

Supplement: S1 Fig — (TIF) [file pcbi.1004512.s001.tif]

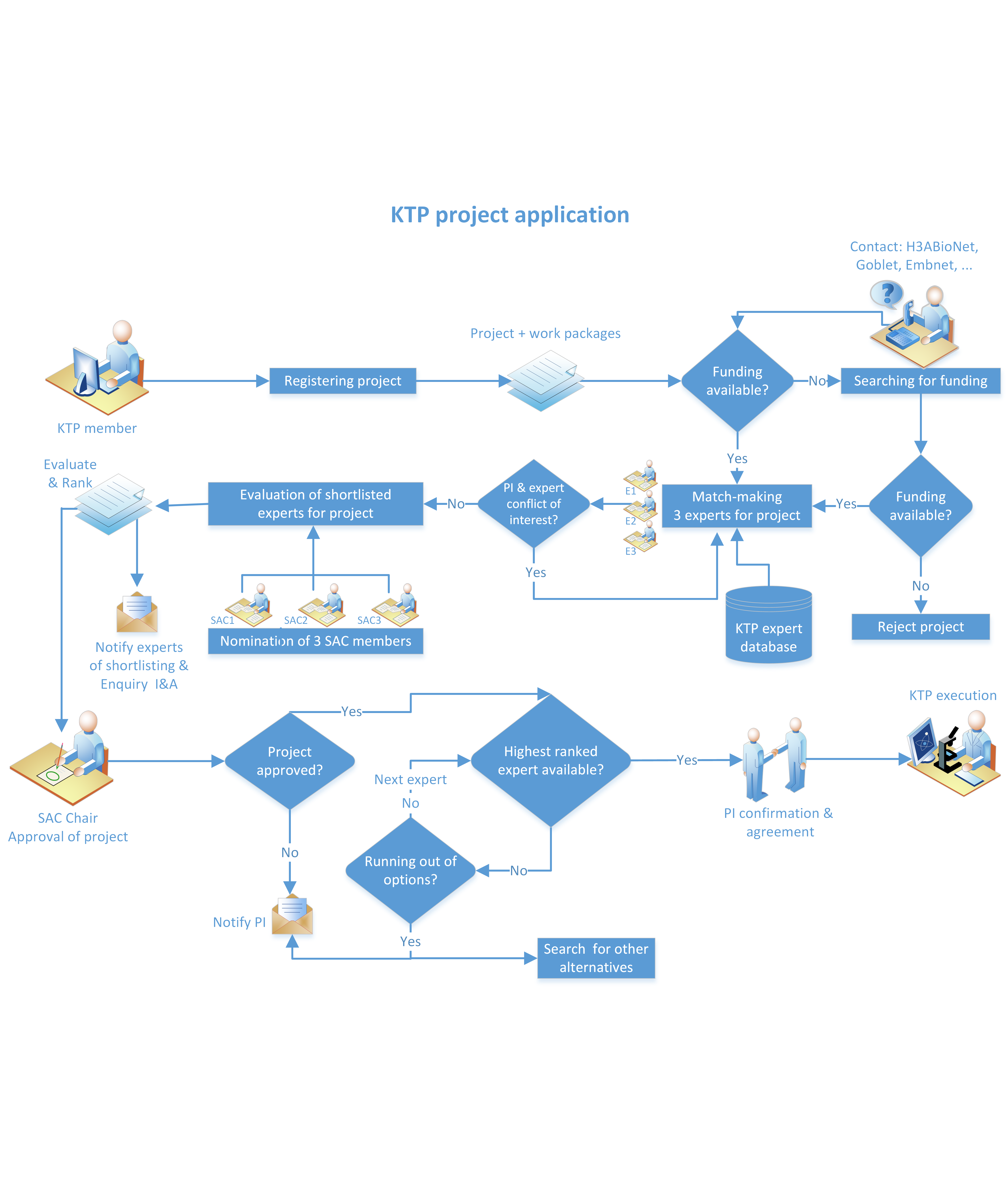

Supplement: S2 Fig — (TIF) [file pcbi.1004512.s002.tif]
