# Supplementary material for: “Broadband” Bioinformatics Skills Transfer with the Knowledge Transfer Programme (KTP): Educational Model for Upliftment and Sustainable Development
Source: PLoS Comput Biol. 2015 Nov 19;11(11):e1004512. doi: 10.1371/journal.pcbi.1004512 (PMC4652891; doi:10.1371/journal.pcbi.1004512)
Supplement: S1 Text — (DOCX) [file pcbi.1004512.s003.docx]

‘Broadband’ Bioinformatics skills transfer with the Knowledge Transfer Programme: educational model for upliftment and sustainable development.

Emile R. Chimusa^2^, Mamana Mbiyavanga^1^, Velaphi Masilela^1^, Judit Kumuthini^1^,

^1^ Centre for Proteomic and Genomic Research, First floor, St. Peter’s Square Mall, Crn Anzio and Main Rd, Observatory, Cape Town, 7925. South Africa.

^2^ Computational Biology Group, Department of Integrative Biomedical Sciences, Institute of Infectious Disease and Molecular Medicine, University of Cape Town, Cape Town, South Africa.

**Correspondence E-mail:** judit.kumuthini@cpgr.org.za

The Induced Brain Drain of Bioinformatics Skills in Africa

With globalization becoming more prominent in the 21^st^ century, a substantial number of African scientists, including bioinformaticians, go abroad because of better job opportunities, competitive salaries, well-resourced institutions and better career development opportunities. This creates a strain on local skilled bioinformaticians while the need for Bioinformatics capabilities continues to increase [23]. However, despite the implemented efforts to supply the increasing demand for Bioinformatics capabilities, there is a considerable need for well-trained specialists in the field of Bioinformatics on the African continent. As a result of what may be perceived as a brain drain of Bioinformatics skills in Africa, an alarming number of countries in Africa have continued to decline in science and technology statistics average [19[,](#_ENREF_17)22[].](#_ENREF_20)

Furthermore, a considerable number of Africa’s university experts have resorted to collaborate with institutions abroad mostly in USA and Europe in order to complete their research and to use the advanced facilities and active infrastructure offered. This has led to a steady loss in expertise, decline in quality of local research output and subsequently inadequate preparation of future bioinformaticians [1]. This has further contributed towards the brain drain as experts seldom return to expand local capacity or transfer knowledge towards capability building. The capacity development approach was initiated on the concept of sending researchers to overseas organizations to acquire critical/technical skills within the field of biomedical studies. Upon their return they are required to share the knowledge and skills with their colleagues in Africa. This model has also applied to Bioinformatics however the shortage of well-trained bioinformaticians in Africa is exacerbated by the lack of readily available mentors, which has resulted in the absence of Bioinformatics training courses within Africa. Many students who have the potential to follow the studies to become bioinformaticians do not receive the necessary insight and mentoring into the field [7].

The general approach to international development for developing countries has shifted over the last few decades, suggesting new approaches to how developing countries could address their problems. One of these approaches is that developing countries should be able to self-identify their problems and collaborate with developed countries to mitigate the problems [20]. Moreover, the field of Bioinformatics exists in a rapidly evolving and continuously redefined environment. This growth necessitates for proportional increase of bioinformaticians to efficiently contribute towards research and new developments, which are of high importance in fields such as life sciences, biotechnology and education in general. Training represents a major element in the transfer of knowledge from clinicians and academics to young generations of scientists [24,25[], in particular in African countries, where efficacious mechanisms are needed to empower the identification of biomedical needs and the design of appropriate solutions. Various initiatives are underway to strengthen the biomedical research capacity in Africa [](#_ENREF_22)1,19,23]. Such initiatives, if well-defined and well implemented, are likely to foster biomedical research. However, current capacity development in biomedical research in Africa is largely based on input from few isolated senior scientists in Africa and many abroad. African scientists are likely to collaborate with their international counterparts for reasons that go beyond scientific compatibility and complementarity. Moreover, collaboration is most successful when there is a basic level of capacity in place to troubleshoot technical problems to achieve the main aim of the projects [1].

The present article is aimed at describing the Knowledge Transfer Programme, an initiative organized by the Centre for Proteomic and Genomic Research (CPGR) to strengthen the Bioinformatics capability building at local institutions where life scientists are in desperate need of skilled bioinformaticians. KTP is a match-making platform that brings together seekers of Bioinformatics expertise with providers of knowledge who are willing to transfer it in a project-based fashion, physically assisting with the development of local trainers. Furthermore, the KTP is a research and education model conceptualised to address these problems in a sustainable manner to stimulate, enhance and strengthen the African Bioinformatics capacity. Its aim is to facilitate the transfer of knowledge and skills from experienced, internationally recognized experts to local scientists in a cost effective manner.

The CPGR, with its KTP partners as hosts, facilitate the process by identifying and evaluating experts, projects and training requirements for potential research associates and local trainees (Fig 1, in the main text). Instead of several associates travelling to well established labs for short periods of time, experts are brought in to interact and conduct high-quality research agendas locally. Experts and associates are brought together to work on relevant projects through which transfer of knowledge and skills is achieved naturally (Fig 1, in the main text). One of the advantages of this approach is that several associates benefit from one expert, while minimising travel and accommodation expenditure (Table 1, in the main text). The programme is designed to be monitored; by the number of local junior and senior Bioinformatics trainers created and number of trainees trained, number of experts in the expert base and the number of projects supported (Table 1, in the main text). The output of each can be evaluated as the number and quality of publications, conference participants, patents or intellectual properties secured.

Match-making Platform

The KTP match-making platform (Fig 1, in the main text) identifies the ideal or most suitable expert for a given project, and is based on the project requirements, similar to how online dating system works. When submitting a project, the (principal investigator) PI will request or identify certain expertise or skills from the KTP skills matrix tree format, which is adopted from (Public Library of Science) PLOS computational biology journal skills ontology. based on matching number of skills requested for a project and skill(s) expert has expert candidates are short-listed from the KTP expert database and subsequently matches are made. The second and critical phase of the match making is done by the Scientific Advisory Committee (SAC) to ensure the project and its work packages can be achieved by the skills and experience experts have. The KTP is organised around six main stakeholders, shown in Table 1 (in the main text). Stakeholders can achieve various levels of mutual benefits, take part in world-class projects in Africa and gain free membership to the KTP network as an endorsed expert by the review committee. Table 1 (in the main text) lists all the benefits to the stakeholders.

Expert Application Process

Reviewing activities of the experts includes assessing and validating their skills, experience and contribution to the scientific community building before admitting them into the system i.e. accepting them as either junior or senior experts. This reviewing process uses a scoring method described below.

S1 Fig illustrates the flow of information during the expert review process: In order to be part of the KTP expert base, a researcher needs first to register as a KTP member and provide as much relevant information as possible in their profile. It is vital to include all relevant skills-set that the candidate considers to be an expert in as the review is done on a per-skill basis. Currently the system supports the skills taxonomy used by the publisher PLOS in its journals to describe the field of an article; we consider this taxonomy robust and with a good coverage for the skills that KTP requires. However the provided tool is not complete, especially in the Bioinformatics area. For this reason the system includes an editor option for skills, which allows options for including suggestions from the KTP members.

Once logged into the system as a KTP member, an application to become a KTP expert can be submitted. The application should include an up to date CV, a motivation letter and any documents that the applicant considers pertinent to demonstrate their expertise. The KTP team will verify submitted information and if the details are satisfactory, a request to review this candidate is sent to 3 of the members of the review committee. After considering the application, each reviewer can provide feedback to the KTP system with his or her observations, and when the 3 reviews are received, the chair of the review committee receives a notification and is requested to make a decision. i.e. tag the candidate as a Senior Expert, Junior Expert or Not an Expert yet. The applicant will be notified of the final decision. S1 Fig is a simplified schematic representation of the expert application process map, which illustrates the steps from application by a KTP to being successfully registered as an expert in the KTP database. The strategy defined in the KTP is to execute the transfer of knowledge using existing projects that require expertise. This ensures that the knowledge offered by the expert is of complete interest to the principal investigator and also attracts the expert community with projects that demonstrate topics of interest.

Project Application Process

The matchmaking process (Fig 1, in the main text) between project and expert starts when a PI (logged into KTP) registers a project in the KTP system. The registration of the project includes basic information about the project (e.g. title, description, objectives) a work package (proposing times and deliverables) and the set of required skills for this project (S2 Fig). Once the PI has approved the availability of funds to bring in an expert, the process can commence. The project goes into stand-by status where a sponsor or other means of funding are explored (S2 Fig). It is after these processes have been completed that matching between the needs of a project and the expert base is done to find experts that are most suitable for the project. A project is listed on the KTP webpage if only its status is “executing” i.e. accepted otherwise, only the PI, PM and SAC Chair can visualize the project and its contents.

The KTP system nominates 3 experts based on the best match of skills required for the proposed project. Following the KTP system nomination 3 members of the SAC proceed to evaluate the project proposal and associated work packages to score the match between the candidate experts and the project. The SAC chair is notified only when the system has captured the 3 evaluations from SAC, he/she proceeds to consolidate the evaluations i.e. if the project is worthy and feasible, a ranking of the best matches between the nominated experts will be created. This ranking is used to define the order in which the experts are consulted in terms of their availability, interest to take part in the project and the skills they have. When an expert agrees to take part in the project, the PI is consulted in order to avoid any conflict of interest, and if all criteria are met the execution stage of the project e.g. relevant work package. If, for any reason, the expert with the highest evaluation score is not available, the next expert with the highest score is appointed to the project. If all the short-listed experts are not available, other options are explored including a new short-listing from the KTP database. In the event, of a matched expert not being identified for the project, other alternatives are explored or ultimately the project is rejected. Once all the administrative agreements and confirmations are done, the project goes live on the KTP platform.

Trainings associated with projects

A trainee can either be a student, researcher or employee from any organisation who is in need of Bioinformatics training. The KTP training programme provides an opportunity for trainees to join a KTP project and experts, in order to create sustainability aimed at proliferating the knowledge transfer across Africa. All KTP projects have Bioinformatics capacity development work packages that trainees can take advantage of by registering through the KTP online system.

The Scoring System

The scoring system is effective for experts or applicants of projects for the KTP according to the KTP's policy for review processes. The scoring system will be used to quantify and qualify the skills provided by the candidate expert (or for eligible criteria on application projects for KTP). Based on the supporting documents submitted by the candidate expert (including CV, supporting letter and other documents), three reviewers chosen from review committees (RC) will score each of the advanced skills. The score will represent an estimate of the level of expertise for the expert. It is therefore critical that candidate experts provide all necessary documentation corroborating their level of expertise for all required fields. In the case of project registration, the score will represent an approximation of the experts’ proficiency and suitability for the project. It must be noted that the score given by a reviewer for a project expert may not fully reflect the level of expertise of the expert candidate, and may therefore be project-specific, rather than expertise specific. This may differ from project to project.

System Implementation

In order to manage all the reasoning behind the different KTP processes, a web system was designed and implemented. The KTP management system has been implemented as a plugin of the widely used open source Content Management System (CMS) called Wordpress (WP), (<https://wordpress.org/>). The KTP has taken advantage of WP plugin architecture that allows to focus on the development of the KTP features, and leave the management of users, the generation of static pages and the management of other contents to WP. Thus, the KTP system is built using PHP + JavaScript programming languages and a MySQL database engine for data storage and manipulation. Through its intuitive user interface, the KTP framework allows users from the WP schema with sufficient access privileges to securely and accurately access KTP entities such as projects, training, host institutions, skills and applications.

As a result of the development process, the website front end allows one to register and login into the system using the intuitive layout of WP. Once logged in, each stakeholder can access and execute their respective tasks, for instance, expert application, evaluation from both the SAC and the RC, registration of projects, etc. The KTP plugin also provides an admin where the manager and administrators of the KTP can assign roles to KTP members (e.g. been part of a committee), edit the skill taxonomy, follow up on expert and project applications, among other KTP specific functionalities. The modularity of both WP and the KTP plugin is ideal to add extra capabilities to the application if required in future.

International links to KTP

International links with Bioinformatics related associations have contributed significantly towards the goal of increasing Bioinformatics capacity and knowledge expansion in Africa- this forms an essential part of the KTP objectives. The interconnected, well-known organisations include GOBLET, Golden Helix foundation, EMBnet, H3ABioNet and H3Africa. These organisations aim to create a conglomerate that comprises of a strong network of Bioinformatics support and expertise, more especially in life sciences fields, that are faced by various challenges to develop and expand research, in certain geographical locations more in Africa. However, efforts have been established, such as the National Institutes of Health and Wellcome Trust (NIH & WT) programmes amongst others in Africa, which funded research organizations located across Africa, where the need for complex/improved scientific skills in the field of Bioinformatics are at the highest need [20,22].

The overarching objectives of “H3Africa” is to empower independent research, foster pan-continental collaboration in biomedical research and to nurture hands-on training to improve research capacity in human hereditary and health. Moreover, facilitation of local researchers and projects while developing Bioinformatics capacity within the African continent to create a sustainable socio economy and to broaden scientific research environment, which will strengthen existing knowledge and sustain knowledge transfer [1,20,24,25].

Further Discussion

Education and skills training are critical to achieving competitive advantage. The level of workforce skills and the ongoing need to update those skills are both rising steadily in new economies that are driven by knowledge. Additionally, workforce requirements have changed due to advancements in ICT (Information and communications technology). Employees are required to have skills affording them to use information to generate knowledge, to engage in collaborative problem solving, make decisions, be self-driven, organized and work independently. Employees need to be team players and respect those from other cultures. These are all 21st century skills, which are rarely covered by formal school curricula [1,26,27]. Therefore preparation is required to create critical mass with essential 21^st^ century skills for the development of expert decision-making and metacognitive strategies i.e. how to proceed when no standard approach seems applicable [23,27]. These are essential for Bioinformatics capacity development in Africa and other parts of the world too. While companies are willing to invest in training people to meet their own requirements, representation in the private companies within the health and biotechnology sectors is lacking. In the African context, these are invaluable skills for the next generation of scientific leaders capable of dealing with unique challenges Africa faces. However, ongoing efforts are in place with a strong focus on skills enhancement and sustainable development in Africa. Moreover, in Bioinformatics the specialized human capital plays a key role in maximizing gains from the big data induced from genetic sequence boom derived from life sciences fields. Additionally, skills development is essential to greater long-term gains for developing and middle income countries in Africa. Therefore, for positive change to occur, the development of skills and institutions that influence economic change needs to be placed at a vocal point for progressive development. Generally building specialized human capital adds value for local suppliers; this creates a large number of direct and indirect jobs, and builds governance capacity as shown by the oil, gas and minerals extractive industries in Africa [27].

Africa has a window of opportunity to broaden and expand the economic benefits derived from their natural capital and transforming this into sustainable economic activities that can generate long-term socio-economic benefits in various fields. This includes petrochemicals and metal mineral extraction in Africa. The lack of specialized expertise has proven to be a major bottleneck, obstructing the potential for more well-paid jobs and home-grown supplier companies [28]. This is a common obstacle also faced within the biotechnology field, where the Bioinformatics skills gap needs to be addressed. This is required to attend to the trend in health expenditure that is fast outgrowing GDP and more importantly the unique population genetic structure present in many countries in Africa.

The KTP is a channel for education and training in Bioinformatics for innovation with the intention of long term capacity development and sustainability for local institutes and companies. Bioinformatics capability building enables the development of trainers and leaders, equipping them with decision-making and metacognitive strategies that indicate how to proceed when no standard approach seems applicable [27]. To remain competitive, workers need to engage in continuous training to update their knowledge and job skills. Employers increasingly require skills in employees such as innovativeness, entrepreneurship, independence of thought and decision making, self-motivation, self-regulation, critical thinking, communication and collaborative problem solving [28]. These can be obtained through skills and expertise being conveyed and subsequently acquired through the knowledge transfer programme.

The KTP is one of the examples of 21^st^ century skills development model, which serves as a platform to solve the problem of shortage in skills within Bioinformatics and its application in the omics field. Therefore, KTP serves as a catalyst for knowledge transfer through skills development undertaken by an expert and trainee through a project based fashion in Africa. As a complement to existing conventional teaching and training methods, KTP enables double cognitive apprenticeship, which refers to direct instruction for cognitive and technical skills, followed by project-based learning in a group in the real world of work. This is supported by trainers and more able peers in response to the increasing demand for Bioinformatics skills levels required for the development of critical mass [29].
